# The spatial context of clinic-reported sexually transmitted infection in Hong Kong

**DOI:** 10.1186/1471-2334-10-275

**Published:** 2010-09-21

**Authors:** Shui-Shan Lee, King-Man Ho, Georgiana MT Cheung

**Affiliations:** 1 Stanley Ho Centre for Emerging Infectious Diseases, The Chinese University of Hong Kong, Hong Kong; 2 Social Hygiene Service, Centre for Health Protection, Department of Health, Hong Kong Special Administrative Region Government, Hong Kong

## Abstract

**Background:**

The incidence and prevalence of sexually transmitted infection (STI) in China has been on the rise in the past decade. Delineation of epidemiologic pattern is often hampered by its uneven distribution. Spatial distribution is often a neglected aspect of STI research, the description of which may enhance epidemiologic surveillance and inform service development.

**Methods:**

Over a one month-period, all first time attendees of 6 public STI clinics in Hong Kong were interviewed before clinical consultation using a standard questionnaire to assess their demographic, clinical and behavioural characteristics. A GIS (geographic information system)-based approach was adopted with mapping performed. The cases attending the clinics in different locations were profiled. A comparison was made between neighbourhood cases (patients living near a clinic) and distant cases (those farther off), by calculating the odds ratio for demographic, behavioural and geographic characteristics.

**Results:**

Of the 1142 STI patients evaluated, the residence locations of 1029 (90.1%) could be geocoded, of which 95.6% were ethnic Chinese and 63.4% male. Geographically only about a quarter lived in the same district as the clinic. STI patients aged 55 or above were more likely to be living in the vicinity of the clinic, located in the same or adjacent tertiary planning unit (a small geographic unit below district level). A majority of patients came from locations a few kilometers from the clinic, the distance of which varies between clinics. Overall, more syphilis cases were reported in patients residing in the same or adjacent tertiary planning unit, while distant cases tended to give a higher risk of inconsistent condom use. There were otherwise no significant clinical and epidemiologic differences between neighbourhood and distant STI cases.

**Conclusions:**

There was no specific relationship between STI and the residence location of patients as regards their clinical and epidemiologic characteristics in the territory of Hong Kong. Older STI patients were however more inclined to attend the nearby STI clinics. Most patients have travelled a variable distance to access the STI service. The relationship between STI clinic cases and distance could be a complex issue intertwined between psychosocial characteristics and STI service coverage.

## Background

Worldwide, sexually transmitted infections (STI) constitute a major group of infections caused by a diversity of microorganisms all sharing the same route of sexual transmission. Globally it was estimated that some 340 million cases of STI had occurred in the year 1999 [1]. Unlike other infectious diseases, STI incidence and prevalence data are scarce because of methodological problems, incomplete reporting, and social stigma associated with its identification [2]. Economic growth and population mobility are other factors that have shaped the transmission dynamics of STI. In China, for example, there was a prolonged period of low STI rate in the past, but the incidence has been increasing lately. From the national surveillance system, there was an over 20% increase of Syphilis between 2006 and 2007, with a reported incidence of 17.16 cases per 100,000 [3]. Delineation of epidemiologic pattern is however hampered by the uneven distribution of STI and its concentration in resource-poor communities, where reliable data are usually limited.

In many countries, clinical reporting continues to be an important means of STI data collection because of the convenience in obtaining and analyzing service statistics. Knowingly, delivery of prompt clinical management with extensive coverage can lead to effective public health outcomes [4]. Clinic-level surveillance can also provide an opportunity for identifying sources of outbreaks through contact tracing [5]. Locating partners of STI patients is, however, a difficult task as it is limited by the private nature of the behaviours behind STI transmission and the unwillingness of patients to disclose relevant details. Epidemiologically, clinic data often reflect just tips of the icebergs, which are in turn affected by health-seeking behaviours in the population. Clearly innovation in the enhancement of STI surveillance is urgently needed [6]. Practically, it would be desirable if STI can be tracked without intruding into the privacy of infected individuals. Apart from exploring new means of surveillance, one strategy is to make good use of available service statistics. Researches have incorporated the assessment of the relatedness of reported cases in a spatial context [7]. The close inter-personal linkage implicated in each sexual transaction lends support to the phenomenon of spatial proximity among STI patients. In this respect, the characterization of the geographic distribution of STI cases could offer a new angle in understanding the epidemiology of the infections on one hand, and the appreciation of service coverage on the other [7,8]. The two dimensions are in fact inter-related, knowing that optimal access to STI treatment is a key to effective control of its spread.

We report here our results of a study conducted in Hong Kong, a small southern Chinese territory of about 1000 Km^2 ^in area bordering Mainland China. With a high population of 7 million, a significant proportion of STI in Hong Kong is managed at the public service. Each year about 13000 STI cases were diagnosed at these public clinics [9]. The yearly incidence of syphilis is less than 15 per 100,000 populations. The situation is unlikely to be a static one as across the border in the city of Shenzhen, the population prevalence of syphilis was high at 0.76% [10]. Assessment of STI cases drawn from public clinics opens up a window of opportunity to assess their spatial pattern, which may improve our characterization of infected patients to better inform control intervention. The common ethnic lineage, the social backgrounds and behavioral profile of STI patients in Hong Kong may be of useful reference in analyzing STI epidemiology in Greater China.

## Methods

In Hong Kong, STI is not a statutory notifiable disease. Epidemiologic surveillance of STI is contributed largely by service data collected by the Government's Social Hygiene Service that is providing free clinical service to over 10,000 patients per year. Operative under the Department of Health, there are currently public STI clinics at 6 geographic locations [11]. The diagnoses of all cases and their demographics are recorded regularly. Each year, over a one-month period, behavioural data are collected from all first time attendees using an assessment form administered face-to-face by clinic staff. This has become one form of enhanced STI surveillance, which has led to the generation of reports on exposure risk to STI/HIV [12]. In 2008 these additional data were anonymised for evaluating the spatial context of STI reports. The assessment tool, in form of a questionnaire (available from authors), was composed of the following parts: (a) demographics - age, gender, marital status, ethnicity, residence location at building level, clinic attended; (b) clinical presentation and specific diagnosis (if established); (c) practice of protected sex in the preceding 3 months and for the last sexual activity with regular, casual and commercial sex partners. Condom use was ranked at four levels: always, frequently, sometimes and never. Specific diagnoses of the following conditions were available from the linked records: syphilis, gonorrhea, non-gonococcal urethritis (NGU), non-specific genital tract infection (NSGI), the latter referring to females who had significant increase in pus cell count but negative Gram stain for gonococcus in their cervical smears obtained on site. Institutional approval for access to the data, the study and reporting of the analysis was sought from Department of Health, Hong Kong Special Administrative Region Government, in compliance with the Personal Data (Privacy) Ordinance. The study conformed to the provision of the Declaration of Helsinki.

In the absence of a standard geocoding system in Hong Kong, georeferenced data were generated manually from residence addresses of respondents and those of the clinics. Digital maps were acquired from the Lands Department of the Hong Kong Special Administrative Region Government. Hong Kong is divided into 18 districts; the population data of each is available through census and by-census. In order to be able to define STI pattern at higher resolution, a smaller geographic unit called tertiary planning unit (TPU) was also used for analyses. There are 399 TPU for planning purpose, each with a different population size. Spatial statistics, including mean centres and 1 SD for cases attending each clinic was determined. Maps were drawn to display the geographic distribution of reported cases, against the background of population statistics from by-census 2006, obtained from the government's Census and Statistics Department. To determine the age effects and STI distribution, all cases were divided into two categories by the normal retirement age: at or below 55, and above 55 years of age. Comparison was made by determining the odds ratio for demographic, behavioural and geographic factors. ArcGIS 9.2 was used for mapping and exploratory spatial analysis. Statistical analysis was performed using Statistical Package for the Social Sciences version 13.0 (SPSS Inc 2004).

## Results

There were altogether 1142 first time attendees during the one-month study period in 2008. Their general characteristics are displayed in Table 1. A total of 1029 (90.1%) could be geocoded, comprising 652 (63.4%) men and 377 (36.6%) women. Almost all (95.6%) were ethnic Chinese. The median age was 34 (range 13 to 97), with male having an older age (median 35, range 16 to 97) than female (median 33, range 15 to 85). Some 13.4% were 55 years old or above, a higher proportion of which were male (male to female ratio: 1.8:1). A majority (87%) did not have a previous diagnosis of STI. A total of 399 (36.3%) were asymptomatic on presentation. More female than male were asymptomatic at presentation. The commonest specific diagnosis was non-gonococcal urethritis (NGU) or non specific genital tract infection (NSGI), accounting for 18.9% of all evaluable cases

**Table 1:** Profile of the study population (n = 1029)

Characteristics			no.	Total (%)
Age (yrs)		Male	female	
	< 25	146	86	232 (22.6)
	25 - 34	179	112	291 (28.3)
	35 - 44	120	94	214 (20.8)
	45 - 54	107	55	162 (15.7)
	55 - 64	53	9	62 (6.0)
	≥ 65	47	21	68 (6.6)
	All	652	377	1029 (100)
				
			**no.**	**(%)**
Male gender			652	(63.4)
				
Ethnicity (n = 1020)	Chinese		975	(95.6)
	Asian non-Chinese		30	(2.9)
	White		13	(1.3)
	Black		2	(0.2)
				
Past history of STI (n = 1026)	Yes		133	(13)
	No		896	(87)
				
District of residence	Same		259	(25.2)
	Different		893	(74.8)
				
Microbiological/specific diagnosis* (n = 1028)	Syphilis		42	(4.1)
	Gonorrhea		52	(5.1)
	NGU/NSGI		193	(18.8)
	Others		754	(73.3)
				
Clinical presentation* (630 symptomatic cases)	Urethral discharge		144	(22.9)
	Dysuria		129	(20.5)
	Ulcer		64	(10.2)
	Growth		206	(32.9)
	Others		133	(24.9)

*multiple diagnoses/presentations in some cases

**Figure 1:**
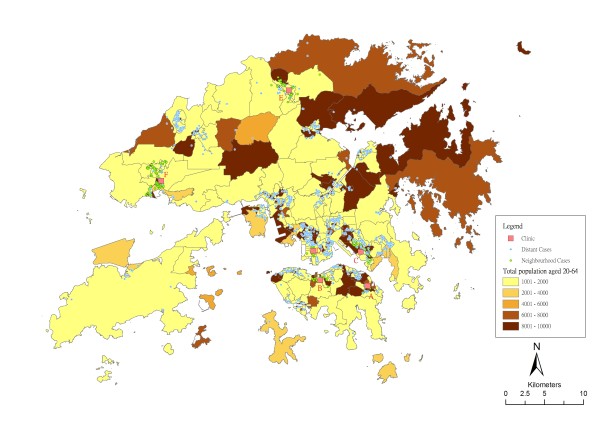
**Geographic distribution of STI patients and Social Hygiene Clinics in Hong Kong, November 2008, against the background of population aged 20 to 64**. The STI cases were further divided into neighbourhood cases (those living in the same or adjacent Tertiary Planning Unit (TPU) as the clinic) and distant cases (those living in other TPU).

**Figure 2:**
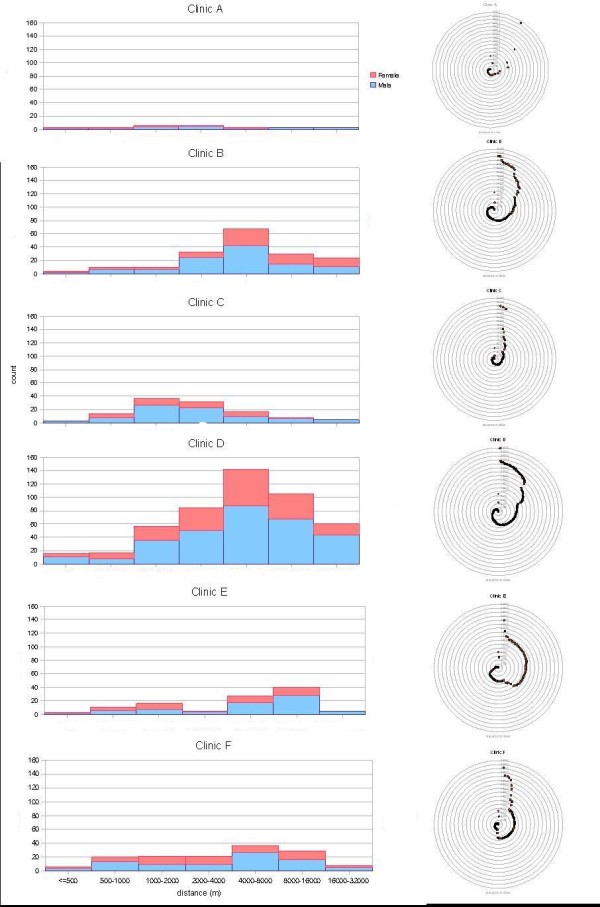
**Distance between residence location of STI patients and the clinics attended, using histogram and net chart**.

There are a total of 6 public STI clinics, 4 located on Hong Kong Island or in Kowloon Peninsula, which are the more densely populated urban areas in the territory of Hong Kong. The other two clinics are in the New Territories' satellite towns, amidst the less populated areas. Almost half (46%) of all cases were recorded at one clinic located in downtown Kowloon (Clinic D), while 4 clinics each accounted for between 10 to 20% of the total caseloads and one at < 2%. Overall about a quarter lived in the same district as the clinic, while 16.6% resided in the same TPU. The distribution of patients and their spatial relationship with the clinics is shown in Figure 1. Patients living in the same or adjacent TPU were defined as "neighbourhood cases", whereas the rest were "distant cases", as illustrated in the map. To protect the identity of the patients, each data point in this map has been randomly redistributed within a radius of 50 metres, which therefore did not reveal the exact residential location of the individuals. Relationship between the number of cases and distance from a clinic is show in figure 2. Interestingly distance decay is not demonstrated. In 3 clinics, a majority of the cases were drawn from a specific distance - 4 to 8 Km for Clinics B and D, 1 - 2 Km for Clinic C. The mean centre of cases for Clinic C was closest to the clinic (1.9 Km), contrasting that of 4 Km or more for the others (Table 2). In one clinic (Clinic E), a bimodal pattern can be seen, reflecting the coverage of two groups of patients, one in the vicinity at between 0.5 Km to 2 Km and the other between 4 and 16 Km. Patients from a broad range of distances went to Clinic A and F. The diversity of the coverage by distance is shown by the radius for 1 standard deviation of total case counts for each clinic, ranging between almost 6 Km to over 9 Km (table 2).

**Table 2:** Distribution of cases for each clinic

Clinic	Distance between mean centre of all cases and the clinic (Km)	Radius of 1 SD (Km)	No. of neighbourhood cases	No. of distant cases
A	3.95	7.67	6	15
B	4.05	9.25	14	162
C	1.93	5.98	39	72
D	4.73	8.94	18	461
E	5.45	8.04	27	78
F	4.06	6.79	67	71

A comparison was made between neighbourhood STI cases and distant cases, the results of which are shown in Table 3. Neighbourhood cases were generally younger (mean = 39.49 vs 36.88 years; p = 0.063) though the difference did not reach statistical significance. Neighbourhood cases were more likely to be 55 years old or older. There were otherwise no other differences between the two in term of demographics. More syphilis cases were however diagnosed amongst neighbourhood case (7% vs 3.4%; OR = 0.48; 95%CI 0.241 - 0.958) (Table 3). Clinical presentation and previous history of STI were not different between the two groups. Condom use was used for evaluating practice of safer sex. Two indicators were used - one on condom use for last sex, and the other on the ranked frequency in the preceding three months (prior to STI diagnosis). Overall, male had a higher tendency to have used condom for casual sex compared to women (Pearson Chi Square test, p < 0.01) in the preceding 3 months. Interestingly, neighbourhood cases gave a slightly lower risk of inconsistent condom use for casual sex compared to distant cases (65% vs 77.8%; OR = 0.465; 95%CI 0.281 - 0.768). The same assessment was repeated after calculating the actual distance in Km between resident and clinic location. Using the median distance as a cutoff, the same age effect could be demonstrated. Distant cases gave an OR of 1.83 (95%CI = 1.04 - 3.24) for being age 55 or above compared to neighbourhood cases. The differences in condom use and specific diagnosis were however not seen in this alternative assessment (results not shown).

**Table 3:** Comparison between STI patients living nearby and at a distance (n = 1029)

Factor		Distant cases^#^	Neighbourhood cases^#^	OR	95% CI
Age*	< = 55	759	140	0.589	0.379 - 0.916
	> 55	99	31		
					
**Gender**	Male	547	105	0.905	0.645 - 1.268
	female	311	66		
					
**Ethnicity**	Non-Chinese	46	8	1.09	0.478 - 2.483
	Chinese	812	163		
					
**past STD**	No	750	146	1.184	0.74 - 1.895
	Yes	108	25		
					
**Presentation**	Asymptomatic	303	67	1.082	0.774 - 1.513
	Symptomatic	555	104		
					
**Condom use**					
	Use for last sex, casual partner	290	52	0.71	0.46 - 1.094
	Inconsistent use^¶^, casual partner*	239	35	0.465	0.281 - 0.768
	Use for last sex, regular partner	220	38	0.824	0.548 - 1.24
	Inconsistent use^¶^, regular partner	199	31	0.739	0.477 - 1.146
					
**Specific diagnosis**					
	Syphilis*	30	12	0.48	0.241 - 0.958
	Gonorrhoea	45	7	1.297	0.575 - 2.926
	NGU/NSGI^†^	155	38	0.772	0.517 - 1.152
					
**TOTAL**	1029	858	171		

^#^Nearby cases - patients living in the same or adjacent TPU (tertiary planning unit); Distant cases - patients living at a distance from the clinic

*p < 0.05

^¶^Inconsistent condom use = not using condom, or using condom infrequently at sexual encounters in the 3 month period prior to the current episode of STI that has led to the clinical consultation.

^†^NGU/NSGI = non-gonococcal urethritis/non-specific genital tract infection

## Discussion

One of the most important observations made in this study was the age difference between neighbourhood cases and distant cases attending public STI clinics in Hong Kong. Our study covered all 6 clinics in Hong Kong which have been providing free drop-in diagnosis and treatment service to anyone who is symptomatic with STI or is concerned about possible infection. It appears that older patients were seen more often in the nearby clinics. This age differential is a cause for concern. The lower mobility of senior citizens could be a reason for the higher concentration of older patients in the vicinity of the clinics. If older patients only go to clinics nearby, then access to STI treatment of those living further away from the clinics is likely to be suboptimal. As treatment offers a window of opportunity for intervention, control of STI spread may also be adversely affected. While the clinics are perceived as conveniently located by younger citizens, our elderly population may have a different view about access. Location of clinics far away from one's residence may be a disincentive for elderly STI patients in seeking treatment. Though STI is a condition of the sexually active, this does not however mean that the infection is confined to young people [13]. Studies have reported a rising incidence of older people with STI [14]. The problem of STI in elderly population can be compounded also by one's low perceived risk of infection, stigma, inconsistent use of condom, and delayed diagnosis [15].

In this spatial study, two other findings were: firstly, the lower prevalence of syphilis, and secondly a higher prevalence of inconsistent condom use in patients living further off. The higher number of syphilis patients in the vicinity of the STI clinics could be related to the administrative arrangement of referring serologically diagnosed cases to a nearby public STI clinic. Serological screening of syphilis is a common practice adopted by some health services to elderly people with neuropsychiatric diseases and any form of motor or sensory deficit. With the rising age of the population, an increasing number of elderly homes are becoming established. The introduction of health screening would uncover otherwise undiagnosed syphilis. This observation may therefore echo the relatively higher proportion of elderly STI patients in the vicinity of the clinics. Our dataset has not included the reasons for each consultation, which therefore did not allow this possible relationship to be determined. Elsewhere, it has also been reported in the literature that the clustering of STI patients could refer to the clustering of social determinants instead of the infection *per se *[16], the relevance of which cannot be substantiated in this study. On the other hand, the marginally higher prevalence of unsafe sexual practice in patients living further from the clinics cannot be explained fully. It can be argued that people living further off may have poorer access to effective safer sex information, or that distant cases represent those with a different demographic profile which may have been associated with the different behavioural pattern. The observations would need to be confirmed by a repeat or expanded study with the objective of determining the association between distance and risk behaviors.

Understandably the spatial variation of STI cases may not necessarily reflect the underlying epidemiologic or behavioural pattern, as it could have been related to the service coverage of individual clinic. It is not surprising to find a higher prevalence of STI in the vicinity of an STI clinic, as patients may be more inclined to present for diagnosis and treatment at a convenient location. Interestingly, our results showed that most of the STI cases did not live near the clinic, even though all clinics are located in residential areas. In some clinics most cases came from a specific distance, implying that they must be residents from another residential area nearby or further away. An inverse relationship between distance and number of cases is not seen. Because of the stigma attached to the disease, some patients may distance themselves from STI services [17]. It is possible that some patients may choose to attend a clinical service unlinked to his/her own residential community. In other patients, they may choose to attend a service near work instead of residence location. It can also be argued that six separate locations cannot provide optimal coverage for STI services, an issue that needs to be further explored when new clinics are planned.

Age differentials aside, our results did not reveal any significant geographic variation in the clinical presentation or diagnoses of STI. This may be related to the pattern of commercial sex industry in the territory. Conventionally, places with discrete foci of commercial sex trade are considered as locations with potential risk of STI spread. In Hong Kong, it is against the law to operate commercial sex trade. While commercial sex workers (CSW) are present, they do not function openly in distinct physical locations [18]. CSW in Hong Kong are apparently quite mobile in their locations of seeking partners and sexual activities. On the other hand, Hong Kong is not just a small area but a place with exceedingly efficient public transport system [19]. Spatial isolation is often not a major issue in term of access to medical facility. It is hard to find STI patients who are geographically isolated because of poor access to public transport. In drawing these and other conclusions on spatial epidemiology, we reckon that the study did carry some limitations. First of all, the analysis was made on patients who have attended the clinic services only during a one-month period in the year 2008. Seasonal patterns, if any, would have distorted the clinical and epidemiologic pictures derived from the study. Secondly, public STI clinics only account for the management of a fraction of all STI cases. Spatial variation of clinical presentations, behaviours and service preference would have been missed if the characteristics of patients not using the Government service differ significantly. As the major service provider using standard protocols for STI management, studies on patients from the clinics do carry an advantage in improving our understanding of STI in the community.

## Conclusions

In conclusion, the public STI clinics in our study are serving the dual role of a patient service on one hand, and a sentinel site for the public health surveillance of STI on the other. Clinic-based surveillance offers a convenient means of collecting regular patient data, the analysis of which could be extrapolated to describe STI epidemiology in a population. Results of such surveillance often provide a snapshot of the situation in a specific place or time, which can also be tracked over time to assess the longitudinal pattern. The spatial context is a less commonly appreciated aspect in STI surveillance. This has prompted us to conduct spatial exploration in order to detect heterogeneity, the results of which, as illustrated in the current study, may carry implications in epidemiologic profiling as well as service coverage.

## Competing interests

The authors declare that they have no completing interests. The opinions and assertions contained herein are private views of the authors and do not reflect those of the Centre for Health Protection, Department of Health, Hong Kong Special Administrative Region Government.

## Authors' contributions

SSL conceptualized the study, conducted the analyses, and wrote the first draft of the manuscript. KMH planned and supervised data collection. GMC coordinated data collection, and both KMH and GMC contributed to the framework for data analysis. All approved the final version of the submitted manuscript.

## Pre-publication history

The pre-publication history for this paper can be accessed here:

http://www.biomedcentral.com/1471-2334/10/275/prepub
